# Application of the Universal Definition of Myocardial Infarction in Clinical Practice in Scotland and Sweden

**DOI:** 10.1001/jamanetworkopen.2024.5853

**Published:** 2024-04-08

**Authors:** Caelan Taggart, Andreas Roos, Erik Kadesjö, Atul Anand, Ziwen Li, Dimitrios Doudesis, Kuan Ken Lee, Anda Bularga, Ryan Wereski, Matthew T. H. Lowry, Andrew R. Chapman, Amy V. Ferry, Anoop S. V. Shah, Anton Gard, Bertil Lindahl, Gustaf Edgren, Nicholas L. Mills, Dorien M. Kimenai

**Affiliations:** 1 British Heart Foundation Centre for Cardiovascular Science, University of Edinburgh, Edinburgh, United Kingdom; 2 Department of Emergency and Reparative Medicine, Karolinska University Hospital, Stockholm, Sweden; 3 Department of Medicine, Clinical Epidemiology Division, Karolinska Institutet, Stockholm, Sweden; 4 Department of Medicine, Karolinska Institutet, Stockholm, Sweden; 5 Department of Non-communicable Disease Epidemiology, London School of Hygiene & Tropical Medicine, London, United Kingdom; 6 Department of Cardiology, Uppsala University, Uppsala, Sweden; 7 Department of Cardiology, Södersjukhuset, Stockholm, Sweden; 8 Usher Institute, University of Edinburgh, Edinburgh, United Kingdom

## Abstract

**Importance:**

Whether the diagnostic classifications proposed by the universal definition of myocardial infarction (MI) to identify type 1 MI due to atherothrombosis and type 2 MI due to myocardial oxygen supply-demand imbalance have been applied consistently in clinical practice is unknown.

**Objective:**

To evaluate the application of the universal definition of MI in consecutive patients with possible MI across 2 health care systems.

**Design, Setting, and Participants:**

This cohort study used data from 2 prospective cohorts enrolling consecutive patients with possible MI in Scotland (2013-2016) and Sweden (2011-2014) to assess accuracy of clinical diagnosis of MI recorded in hospital records for patients with an adjudicated diagnosis of type 1 or type 2 MI. Data were analyzed from August 2022 to February 2023.

**Main Outcomes and Measures:**

The main outcome was the proportion of patients with a clinical diagnosis of MI recorded in the hospital records who had type 1 or type 2 MI, adjudicated by an independent panel according to the universal definition. Characteristics and risk of subsequent MI or cardiovascular death at 1 year were compared.

**Results:**

A total of 50 356 patients were assessed. The cohort from Scotland included 28 783 (15 562 men [54%]; mean [SD] age, 60 [17] years), and the cohort from Sweden included 21 573 (11 110 men [51%]; mean [SD] age, 56 [17] years) patients. In Scotland, a clinical diagnosis of MI was recorded in 2506 of 3187 patients with an adjudicated diagnosis of type 1 MI (79%) and 122 of 716 patients with an adjudicated diagnosis of type 2 MI (17%). Similar findings were observed in Sweden, with 970 of 1111 patients with adjudicated diagnosis of type 1 MI (87%) and 57 of 251 patients with adjudicated diagnosis of type 2 MI (23%) receiving a clinical diagnosis of MI. Patients with an adjudicated diagnosis of type 1 MI without a clinical diagnosis were more likely to be women (eg, 336 women [49%] vs 909 women [36%] in Scotland; *P* < .001) and older (mean [SD] age, 71 [14] v 67 [14] years in Scotland, *P* < .001) and, when adjusting for competing risk from noncardiovascular death, were at similar or increased risk of subsequent MI or cardiovascular death compared with patients with a clinical diagnosis of MI (eg, 29% vs 18% in Scotland; *P* < .001).

**Conclusions and Relevance:**

In this cohort study, the universal definition of MI was not consistently applied in clinical practice, with a minority of patients with type 2 MI identified, and type 1 MI underrecognized in women and older persons, suggesting uncertainty remains regarding the diagnostic criteria or value of the classification.

## Key Points

**Question:**

How is the universal definition of myocardial infarction (MI), which differentiates type 1 (atherothrombosis) from type 2 (oxygen supply-demand imbalance) MI, applied in clinical practice?

**Findings:**

In this cohort study of 50 356 patients, few patients meeting the diagnostic criteria for type 2 MI received a clinical diagnosis of MI in practice, and type 1 MI was underdiagnosed in women and older people.

**Meaning:**

These findings suggest that uncertainty remains regarding the diagnostic criteria or value of the universal definition of MI.

## Teaser

This cohort study evaluates the application of the universal definition of myocardial infarction (MI) in consecutive patients with possible MI across 2 health care systems in Scotland and Sweden.

## Introduction

The universal definition of myocardial infarction (UDMI) is endorsed by the World Health Organization and encourages the use of standard criteria for the diagnosis of MI worldwide.^1,2,3^ In 2007, a classification into subtypes was introduced that recognized there are different underlying pathophysiological mechanisms of MI.^4^ Type 1 MI due to coronary atherothrombosis is well established in practice, and clear guidance is available for management and treatment of this condition.^2^ In contrast, type 2 MI due to oxygen supply-demand imbalance may be triggered by multiple conditions, and the implications of this diagnosis in practice are less certain.^5,6,7,8,9^

Outcomes for patients with type 2 MI are variable and are often worse than for patients with type 1 MI.^10,11,12,13,14^ Patients with type 2 MI often are older and have more comorbidities,^15^ but differences in outcome may also reflect uncertainty in practice and variation in the management of patients with type 2 MI. It is currently unclear whether the diagnostic criteria proposed by the UDMI are consistently applied in clinical practice.

In consecutive patients with possible MI presenting to secondary or tertiary care hospitals across 2 different countries, we evaluated the proportion of patients with a clinical diagnosis of MI recorded in the hospital records who had type 1 and type 2 MI as adjudicated by an independent panel according to the UDMI. We compared the characteristics and outcomes in patients with and without a clinical diagnosis MI.

## Methods

This cohort study was conducted according to the Declaration of Helsinki and approved by local research ethics committees in Scotland and Sweden. These approvals did not require individual patient consent, as both Scotland and Sweden allow for research to take place without consent in limited circumstance.^16,17^ All data were linked and deidentified within an approved secure data environment (DataLoch). We adhered to the Strengthening the Reporting of Observational Studies in Epidemiology (STROBE) reporting guideline.

### Study Populations

For the cohort from Scotland, we used data from High-Sensitivity Troponin in the Evaluation of Patients With Suspected Acute Coronary Syndrome (High-STEACS) trial.^18^ High-STEACS evaluated the implementation of a high-sensitivity cardiac troponin I assay in consecutive patients with suspected MI across 10 secondary and tertiary care hospitals in Scotland between 2013 and 2016. Patients were eligible for inclusion if they presented with suspected MI and had paired contemporary and high-sensitivity cardiac troponin measurements. Patients were excluded if they had been admitted previously during the trial period or were not residents of Scotland.

For the cohort from Sweden, we used data from a prospective observational cohort study of patients with suspected MI who attended the emergency department (ED) of Karolinska University Hospital in Stockholm between 2011 and 2014.^19^ All patients older than 25 years attending the ED with chest pain in whom at least 1 measurement of high-sensitivity cardiac troponin was available were eligible for inclusion.

For this study, we excluded patients where adjudicators determined there was insufficient clinical information to enable adjudication of diagnosis. This was a result of not having access to linked records describing presentation to ED or hospital admission. For the cohort from Scotland, we also excluded patients admitted during the validation phase of the trial, as care was not guided by a high-sensitivity troponin assay.

### Adjudicated Diagnosis of MI

All patients with evidence of myocardial injury were adjudicated and classified according to the fourth UDMI (eMethods in Supplement 1). In the cohort from Scotland, myocardial injury was defined as any high-sensitivity troponin I concentration above the sex-specific 99th percentile threshold. Cardiac troponin was measured using the ARCHITECT*_STAT_* high-sensitive troponin I assay (Abbott Diagnostics), with the 99th percentile defined as 34 ng/L in men and 16 ng/L in women (to convert to nanograms per milliliter, multiply by 0.001).^20^ In the cohort from Sweden, myocardial injury was defined as any high-sensitivity cardiac troponin T concentration above the uniform 99th percentile threshold. Cardiac troponin was measured using the Elecsys high-sensitivity troponin T (Roche Diagnostics), with the 99th percentile defined as 14 ng/L.^21^

### Clinical Diagnosis of MI

Clinical diagnoses were listed by the consultant overseeing patients care on hospital discharge letter. Letters were reviewed by a team of professional coders at each hospital site who classified clinical diagnoses according to the *International Statistical Classification of Diseases and Related Health Problems, Tenth Revision *(*ICD-10*) system. The classification was then reviewed and finalized by Public Health Scotland and the original patient’s consultant to create the Scottish Morbidity Record and Swedish Patient Register. For primary analysis, we identified patients with MI if an I21 or I22 code was listed in the first or any subsequent position.

### Clinical Outcomes

For the cohort from Scotland, regional and national registries were used to collect data on outcomes, and all subsequent hospital admissions with myocardial injury or deaths were adjudicated by clinicians blinded to the index diagnosis and study phase as previously described.^18^ For the cohort from Sweden, the Swedish National Patient Register and Causes of Death Register were used to identify subsequent hospital admissions and cause-specific deaths. For this analysis, the primary outcome was subsequent MI after index hospital presentation (*ICD-10* codes I22-I22) or cardiovascular death at 1 year. Secondary outcomes included subsequent MI, cardiovascular death, or all-cause death at 1 year.

### Statistical Analysis

We calculated sensitivity, specificity, negative predictive value, and positive predictive value of clinical diagnosis of MI for any adjudicated diagnosis of MI and for type 1 and type 2 MI separately. The 95% CIs were determined using a bayesian approach by sampling from a binomial likelihood with noninformative Jeffreys prior (both β-distribution shape parameters = 0.5). We used the Cohen κ to evaluate concordance between clinical and adjudicated diagnosis of MI.

In patients with an adjudicated diagnosis of type 1 or type 2 MI, we conducted univariable and multivariable logistic regression analyses to quantify the association between clinical characteristics and the odds of a clinical diagnosis of MI. We adjusted for age, sex, hemoglobin and estimated glomerular filtration rate (eGFR) at presentation, peak cardiac troponin concentrations as well as a previous diagnosis of ischemic heart disease, previous cerebrovascular disease, diabetes, previous heart failure hospitalization, and myocardial ischemia. To achieve a normal distribution, we log_2_ transformed cardiac troponin.

We estimated the cumulative incidence of the primary outcome, and group comparisons were made using log-rank test. Cox regression analyses were conducted to evaluate the association between receiving a clinical diagnosis of MI and the primary outcome. In multivariable analyses, we adjusted for age and sex and subsequently added the covariates to the model used in logistic regression analysis. Noncardiovascular death was considered a competing risk.

We conducted several exploratory analyses. We evaluated whether the position of *ICD-10* code within the hospital record from the first up to the sixth position influenced agreement with adjudicated diagnosis. Second, we assessed agreement between clinical and adjudicated diagnoses in patients with ST-segment elevation (STEMI) and non-STEMI separately. Finally, we evaluated differences between patients who did and did not receive a clinical diagnosis of MI in association with secondary outcomes.

Multiple imputation by chained equations was used to impute missing covariate data using other clinical characteristics and outcomes. All analyses were undertaken between August 2022 and February 2023 using R software version 3.6.3 (R Project for Statistical Computing). Comparisons with a 2-sided *P* < .05 were considered statistically significant.

## Results

### Study Populations

**Figure 1.**
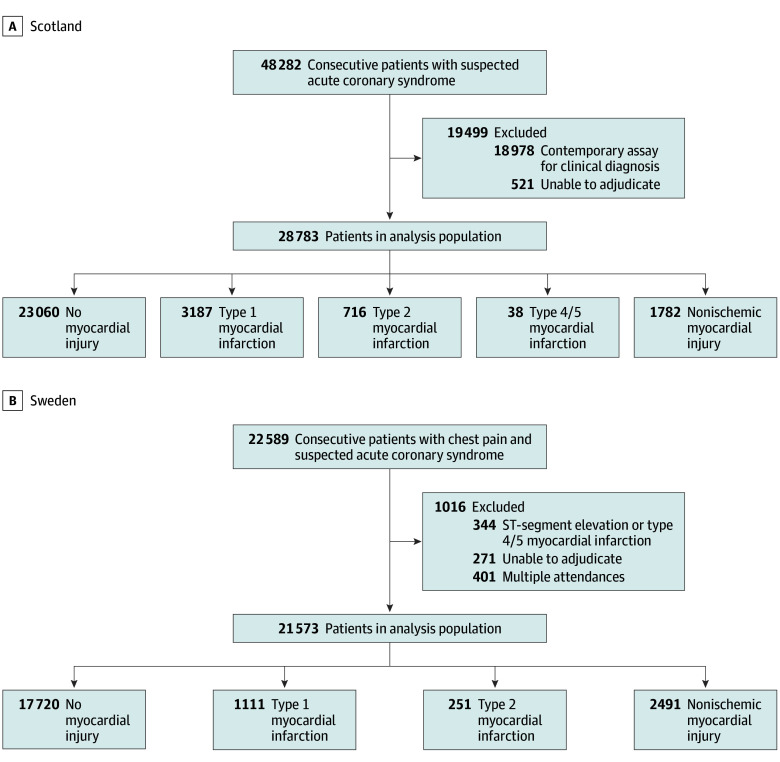
Flowcharts of Identification of Patients in Scottish and Swedish Cohorts

A total of 50 356 patients were assessed. The cohort from Scotland included 28 783 patients (15 562 men [54%]; mean [SD] age, 60 [17] years), and the cohort from Sweden included 21 573 patients (11 110 men [51%]; mean [SD] age, 56 [17] years) (Figure 1; eTable 1 in Supplement 1). In Scotland, 3187 patients (11%) had an adjudicated diagnosis of type 1 MI and 716 patients (3%) had an adjudicated diagnosis of type 2 MI (Table 1). In Sweden, 1111 patients (5%) had an adjudicated diagnosis of type 1 MI and 251 patients (1%) had an adjudicated diagnosis of type 2 MI (Table 1).

**Table 1.** Clinical Characteristics of Patients With and Without a Clinical Diagnosis of MI in Scotland and Sweden

Adjudicated diagnosis	Scotland^a^				Sweden			
	Type 1 MI		Type 2 MI		Type 1 MI		Type 2 MI	
	With clinical diagnosis (n = 2506)	Without clinical diagnosis (n = 681)	With clinical diagnosis (n = 122)	Without clinical diagnosis (n = 594)	With clinical diagnosis (n = 970)	Without clinical diagnosis (n = 141)	With clinical diagnosis (n = 57)	Without clinical diagnosis (n = 194)
Age, mean (SD), y	67 (14)	71 (14)	74 (15)	74 (14)	69 (13)	70 (12)	76 (14)	71 (13)
Sex								
Female	909 (36)	336 (49	63 (52)	317 (53)	298 (31)	54 (38)	26 (46)	102 (53)
Male	1597 (64)	345 (51)	59 (48)	277 (47)	672 (69)	87 (62)	31 (54)	92 (47)
Chest pain at presentation	2282 (91)	554 (81)	93 (76)	426 (72)	970 (100)	141 (100)	57 (100)	194 (100)
Past medical history								
Aspirin	752 (30)	267 (39)	56 (46)	229 (39)	355 (37)	72 (51)	26 (46)	75 (39)
Lipid-lowering therapy	1096 (44)	381 (56)	64 (52)	329 (55)	305 (31)	67 (48)	16 (28)	72 (37)
ACE inhibitor or ARB blocker	941 (38)	305 (45)	56 (46)	250 (42)	396 (41)	72 (51)	30 (53)	101 (52)
β-Blocker	702 (28)	252 (37)	48 (39)	239 (40)	381 (39)	77 (55)	30 (53)	111 (57)
MI	250 (10)	123 (18)	24 (20)	72 (12)	158 (16)	34 (24)	8 (14)	40 (21)
Ischemic heart disease	591 (24)	282 (41)	55 (45)	211 (36)	229 (24)	48 (34)	16 (28)	52 (27)
Cerebrovascular disease	155 (6)	81 (12)	19 (16)	61 (10)	62 (6)	9 (6)	5 (9)	14 (7)
Diabetes	395 (16)	107 (16)	19 (16)	68 (11)	182 (19)	31 (22)	14 (25)	42 (22)
HF hospitalization	290 (12)	137 (20)	35 (29)	138 (23)	111 (11)	24 (17)	16 (28)	38 (20)
Revascularization	244 (10)	112 (16)	21 (14)	55 (7)	173 (18)	42 (30)	12 (21)	43 (22)
Electrocardiographic findings								
Myocardial ischemia	1165 (51)	145 (24)	58 (51)	202 (36)	265 (27)	36 (26)	18 (32)	48 (25)
ST segment elevation	599 (26)	41 (7)	11 (10)	15 (3)	NA	NA	NA	NA
ST depression	511 (22)	75 (13)	42 (37)	140 (25)	NA	NA	NA	NA
T wave inversion	411 (18)	89 (15)	22 (19)	83 (15)	NA	NA	NA	NA
Heart rate, mean (SD), bpm	78 (20)	79 (20)	100 (28)	106 (35)	76 (17)	76 (17)	94 (33)	95 (31)
SBP, mean (SD), mm Hg	143 (28)	142 (28)	134 (31)	132 (30)	154 (28)	151 (27)	151 (38)	138 (33)
Hemoglobin, mean (SD), g/dL	13.9 (1.9)	13.2 (2.0)	12.2 (3.3)	13.1 (2.2)	13.9 (1.7)	13.6 (1.8)	12.1 (3.1)	12.8 (2.5)
eGFR, mean (SD), mL/min/1.73 m^2^	76 (25)	67 (26)	63 (28)	64 (25)	75 (23)	68 (25)	63 (27)	66 (24)
Peak troponin I, median (IQR), ng/L	1928 (269-11 822)	84 (37-865)	1205 (266-3910)	103 (44-292)	NA	NA	NA	NA
Peak troponin T, median (IQR), ng/L	NA	NA	NA	NA	217 (65-679)	38 (23-108)	139 (77-460)	60 (28-131)

Abbreviations: ACE, angiotensin-converting enzyme; ARB, angiotensin receptor blocker; bpm, beats per minute; eGFR, estimated glomerular filtration rate; HF, heart failure; MI, myocardial infarction; NA, not applicable; SBP, systolic blood pressure.

SI conversion factor: To convert hemoglobin to grams per liter, multiply by 10; troponin to nanograms per milliliter, multiply by 0.001.

^a^ Missing values were less than 5% if applicable in both cohorts, except for SBP (1509/3903 [39%]), heart rate (740/3903 [19%]), and electrocardiographic findings (359/3903 [9%)] in the Scottish cohort.

### Clinical Diagnosis of MI

**Figure 2.**
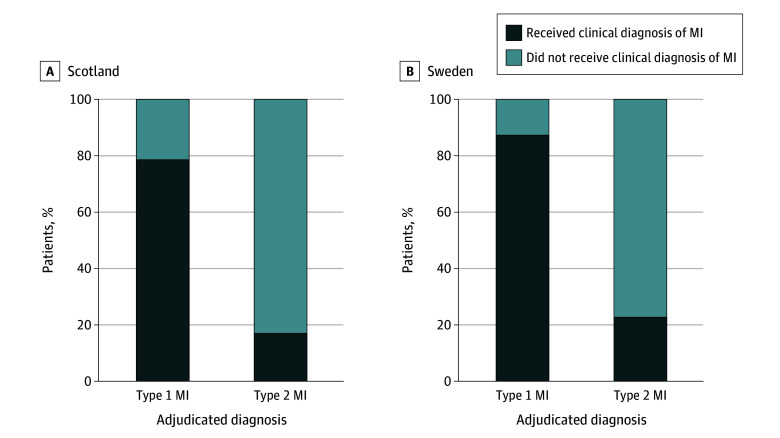
Proportion of Patients With an Adjudicated Diagnosis of Type 1 and Type 2 Myocardial Infarction (MI) With and Without a Clinical Diagnosis of MI in Scotland and Sweden

A clinical diagnosis of MI was recorded in 2657 of 3941 patients (67%) with any adjudicated MI diagnosis in Scotland (κ = 0.757) and 1027 of 1362 patients (75%) with any adjudicated MI diagnosis in Sweden (κ = 0.839) (Table 2). In Scotland, a clinical diagnosis of MI was recorded in 2506 patients (79%) with an adjudicated type 1 MI diagnosis and 122 patients (17%) with an adjudicated type 2 MI diagnosis (Figure 2). Findings were consistent in the cohort from Sweden, with 970 patients (87%) with a type 1 MI diagnosis and 57 patients (23%) with a type 2 MI diagnosis receiving a clinical diagnosis of MI. A small number of patients received a clinical diagnosis of MI but not an adjudicated diagnosis of any type of MI (Scotland: 169 patients [0.4%]; Sweden: 33 patients [0.2%]). No differences were observed in the proportion of patients receiving a clinical diagnosis of MI over time *(*eFigure 1 in Supplement 1).

**Table 2.** Diagnostic Performance of a Clinical Diagnosis of MI in the Hospital Record Stratified by the Universal Definition

Adjudicated diagnosis	Clinical diagnosis of MI in Scotland			Clinical diagnosis of MI in Sweden		
	Any (n = 3941)	Type 1 (n = 3187)	Type 2 (n = 716)	Any (n = 1362)	Type 1 (n = 1111)	Type 2 (n = 251)
True positives	2657	2506	122	1027	970	57
False positives	169	320	2704	33	90	1003
True negatives	24 673	25 276	25 363	20 178	20 372	20 319
False negatives	1284	681	594	335	141	194
Sensitivity (95% CI)	67.4 (65.9-68.8)	78.6 (77.2-80.0)	17.1 (14.4-20.0)	75.4 (73.1-77.6)	87.3 (85.3-89.2)	22.8 (17.9-28.2)
Specificity (95% CI)	99.3 (99.2-99.4)	98.7 (98.6-98.9)	90.4 (90.0-90.7)	99.8 (99.8-99.9)	99.6 (99.5-99.6)	95.2 (95.0-95.6)
NPV (95% CI)	95.1 (94.8-95.3)	97.4 (97.2-97.6)	97.8 (97.5-97.9)	98.4 (98.2-98.5)	99.3 (99.2-99.4)	99.1 (98.9-99.2)
PPV (95% CI)	94.0 (93.1-94.9)	88.7 (87.5-89.8)	4.3 (4.0-5.1)	96.8 (95.7-97.8)	91.5 (89.7-93.1)	5.4 (4.1-6.9)
Cohen κ	0.757	0.814	0.030	0.839	0.888	0.069

Abbreviations: MI, myocardial infarction; NPV, negative predictive value; PPV, positive predictive value.

#### Characteristics of Patients With an Adjudicated Diagnosis of Type 1 and Type 2 MI Not Identified in Clinical Practice

In Scotland, patients with an adjudicated diagnosis of type 1 MI without a clinical diagnosis of MI were more likely to be women (336 patients [49%] vs 909 patients [36%]; *P* < .001) and to be older (mean [SD] age, 71 [14] years vs 67 [14] years; *P* < .001) than patients who received a clinical diagnosis of MI. In contrast, no differences were observed in the clinical characteristics between patients with an adjudicated diagnosis of type 2 MI with and without a clinical diagnosis of MI. After adjustment for cardiovascular comorbidities and clinical features, age and sex were no longer associated with a clinical diagnosis of MI (eTable 2 in Supplement 1). In Scotland, higher cardiac troponin concentrations were associated with a clinical diagnosis of MI, and a similar association was found in Sweden (Table 1).

#### Management and Clinical Outcomes of Patients With an Adjudicated Diagnosis of Type 1 and Type 2 MI Not Identified in Clinical Practice

For both type 1 and type 2 MI, the frequency of coronary angiography at 30 days was higher in patients with a clinical diagnosis compared with patients without a clinical diagnosis in Scotland (type 1: 1883 patients [75%] vs 176 patients [26%]; *P* < .001; type 2: 25 patients [20%] vs 57 patients [10%]; *P* < .001) (eTable 3 in Supplement 1) . Findings were consistent in Sweden (eTable 3 in Supplement 1). Similarly in both countries, use of secondary prevention was higher in patients with a clinical diagnosis of MI for both type 1 and type 2 MI.

**Figure 3.**
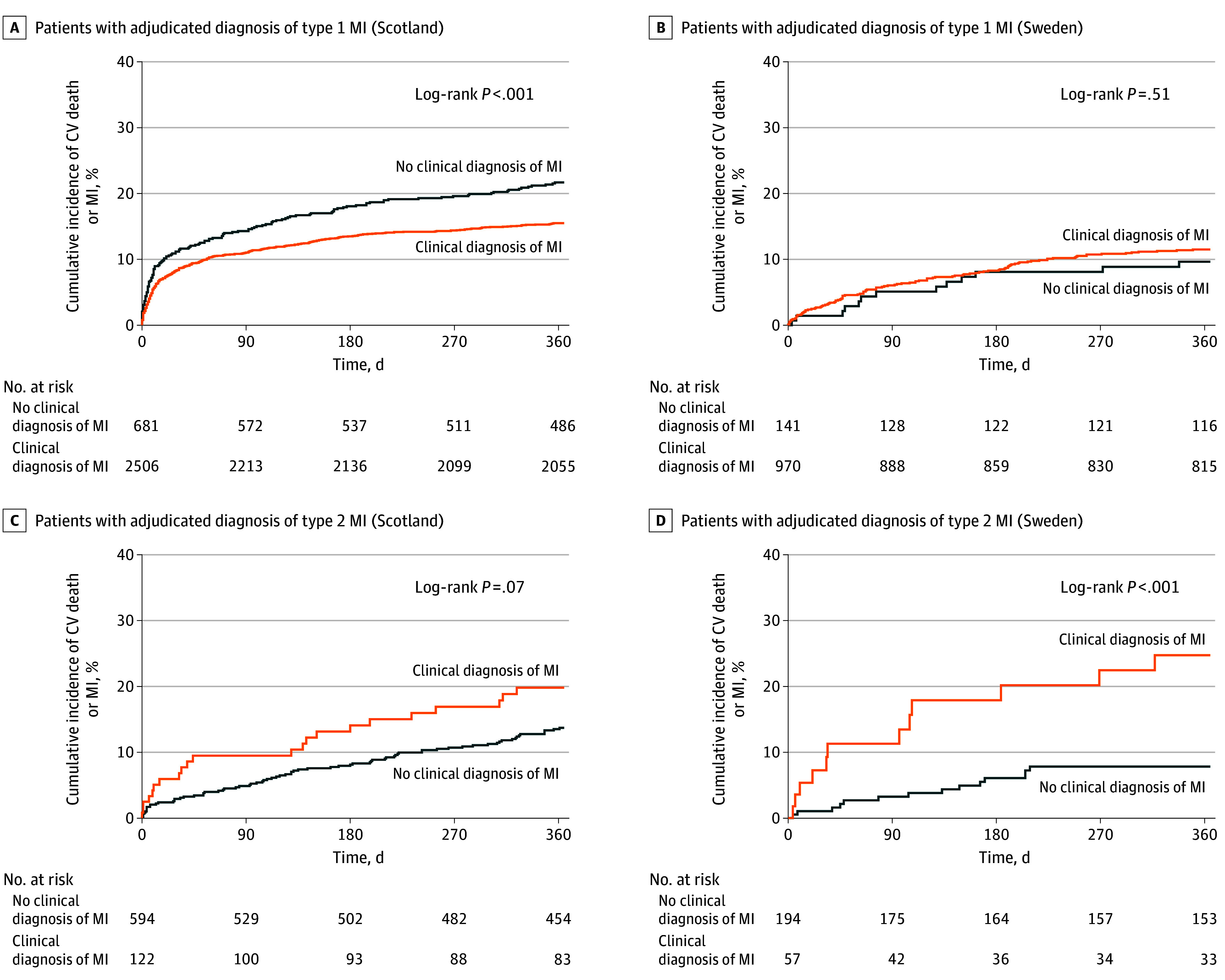
Cumulative Incidence of Myocardial Infarction (MI) or Cardiovascular (CV) Death at 1 Year in Patients With an Adjudicated Diagnosis of MI Stratified According Receipt of a Clinical Diagnosis of MI

In Scotland, the primary outcome occurred more often in patients with an adjudicated diagnosis of type 1 MI who did not have a clinical MI diagnosis compared with those with a clinical diagnosis of MI (29% vs 18%; *P* < .001) (Figure 3). The primary outcome for patients with type 1 MI with and without a clinical diagnosis in Sweden was similar (18% vs 16%; *P* = .51). In contrast, patients with an adjudicated diagnosis of type 2 MI who did not have a clinical diagnosis of MI had a lower cumulative incidence of the primary outcome compared with patients with a clinical diagnosis (Scotland: 24% vs32%; *P* < .001; Sweden: 21% vs 42%; *P* < .001). Differences between groups were attenuated after adjustment for sex, age, and other known cardiovascular risk factors (eTable 4 in Supplement 1).

### Exploratory Analyses

For primary analysis, we identified patients with MI if an I21 or I22 code was listed in any position. Findings were consistent in our exploratory analysis when we restricted the diagnostic code for MI to the first position, with excellent agreement between adjudicated and clinical diagnosis for type 1 MI but not for type 2 MI (eTable 5 in Supplement 1). Diagnostic accuracy and outcomes were similar in the cohort from Scotland when patients with STEMI were excluded (eTable 6 and eFigure 2 in Supplement 1). A clinical diagnosis of MI was recorded in 93% of all patients with an adjudicated diagnosis of type 1 or type 2 STEMI in Scotland, and neither sex nor age were associated with receiving a clinical diagnosis in this subgroup ***(***eTable 7 in Supplement 1). As for primary outcome, we observed similar findings for our secondary outcomes at 1 year in both cohorts (eFigures 3-6 in Supplement 1).

## Discussion

In this cohort study among consecutive patients across 2 different countries, we evaluated how the UDMI has been applied in practice. Our main finding was that fewer than 1 in 5 patients who met the diagnostic criteria for type 2 MI received the diagnosis in practice. In contrast, 4 in 5 patients with an adjudicated diagnosis of type 1 MI were identified in practice. Patients meeting the diagnostic criteria for type 1 MI but not classified as having MI in practice were more likely to be women, be older, or have had a small increase in cardiac troponin. Despite these patients being less likely to undergo coronary angiography or to receive secondary prevention, they were at similar or higher risk of subsequent MI or cardiovascular death than those with a clinical diagnosis of MI.

Our study has several strengths. First, we evaluated this research question in 2 prospective cohort studies from countries with universal health care. In both countries, hospital discharge codes are used for public health surveillance rather than financial reimbursement, reducing the risk of ascertainment bias. Second, in both cohorts the criterion standard was adjudicated according to UDMI. Third, our study comprised consecutive patients with possible MI evaluated using cardiac troponin I and T; therefore, our findings are likely to apply to health care systems using either assay.

Only a minority of patients who met the diagnostic criteria for type 2 MI received this diagnosis in clinical practice. This observation is consistent with findings from registries, which have reported that the diagnosis of MI was recorded in one-third of patients with type 2 MI identified by adjudication.^22^ Patients with type 2 MI were more often treated outside the coronary care unit, which may have contributed to misclassification.^22^ Together these studies suggest considerable uncertainty as to how to apply the diagnostic criteria for type 2 MI in practice. The current classification encompasses a broad range of patients, from those with coronary mechanisms of MI to those without any underlying coronary artery disease.^8,15,23,24^ Furthermore, the diagnosis requires evidence of symptoms or signs of myocardial ischemia, which can be more difficult to ascertain in patients presenting with another acute condition. As such, alternative classifications have been proposed with more objective diagnostic criteria recognizing that MI can arise spontaneously, secondary to another condition or as a complication of a cardiac procedure.^25^

Our findings raise another more fundamental question about the value of a diagnosis of type 2 MI in practice. The classification of any disease should inform the patient of their prognosis and guide the approach to treatment.^26^ Currently we lack evidence-based recommendations for management and treatment of patients with type 2 MI.^9^ While there is little doubt that patients with type 2 MI are at risk of major cardiovascular events,^10,24^ until there are well-defined management and treatment implications for patients with this condition, it is likely that clinicians will prioritize management of the primary presenting condition and be less likely to recognize type 2 MI in practice.

In Scotland, patients meeting the diagnostic criteria for type 1 MI but not classified as having MI in practice were more likely to be women or to be older. However, these observed differences were not independent of differences in other clinical features, suggesting that the presence of comorbidity or magnitude of troponin increase is more likely to influence clinical diagnosis than age and sex per se. Our observations are consistent with previous work demonstrating sex- and age-associated differences in diagnosis of MI^27,28,29^ and underline the need to increase awareness to prevent inequalities in care.

Misclassified patients with type 1 and type 2 MI underwent fewer investigations and were less likely to receive preventative therapies. The risk of future MI or cardiovascular death was higher or as high in patients with type 1 MI who did not receive a diagnosis of MI in practice. While we observed that patients with type 1 MI who were not diagnosed with MI in practice in Scotland remained at higher risk after adjustment for risk factors, we did not observe this in Sweden. This may be a consequence of the reliability of the primary outcome measure between 2 cohorts. All subsequent events where patients reattended with evidence of myocardial injury were adjudicated in Scotland, while we relied on *ICD-10* coding to identify events in Sweden. Variation could also be due to differences in the troponin assay, diagnostic pathway, application of sex-specific criteria or the use of cardiac investigations. In contrast, we observed that patients with type 2 MI who did not receive a clinical diagnosis of MI were at lower risk of future events than those recognized in practice in both countries. It appears that clinicians are more likely to use the term MI in the setting of myocardial oxygen supply demand mismatch if they recognize the patient to be at particularly high risk of future cardiovascular events.

Hospital discharge codes for MI are based on *ICD-10* rather than the UDMI. Unfortunately, *ICD-10* does not recognize the 5 subtypes of MI described in the UDMI. While some health care systems or insurance providers have recently introduced an additional digit for *ICD-10* codes to identify patients with type 2 MI (I21.A1) this is not universally applied and the accuracy of these codes is unknown.^30,31,32^

### Limitations

Our study has several limitations. First, no data on race or ethnicity were available. On a national level, both cohorts included predominantly a White population, which may limit the generalizability of our findings. Second, we could not determine whether misclassification was a result of the responsible clinician not applying the diagnosis or whether it arose due to errors in hospital discharge coding. Third, we were not able to evaluate accuracy of the new *ICD-10* diagnostic code for type 2 MI (I21.A1), which was introduced for billing purposes in the US in 2017, as this code is not used in either United Kingdom or Sweden.

## Conclusions

This cohort study found that the diagnostic classification proposed by the UDMI was not consistently applied in clinical practice. Our findings suggest uncertainty remains regarding the diagnostic criteria or value of this classification in practice.

## Notes

Supplement 1. **eMethods.** Adjudication Process **eFigure 1.** Proportion of Patients With an Adjudicated Diagnosis of Type 1 Myocardial Infarction and Type 2 Myocardial Infarction Who Had a Clinical Diagnosis of Myocardial Infarction in the Hospital Record Over 6-Month Blocks in Scotland and Sweden **eFigure 2.** Cumulative Incidence of Cardiovascular Death or Myocardial Infarction in All Universal Definition Subtypes of Myocardial Infarction With No ST Elevation and ST Elevations Stratified According to Those With a Clinical Diagnosis and Without a Clinical Diagnosis of Myocardial Infarction **eFigure 3.** Cumulative Incidence of All Cause Death in All Myocardial Infarctions in Scotland and Sweden Stratified According to Those With a Clinical Diagnosis and Without a Clinical Diagnosis of Myocardial Infarction **eFigure 4.** Cumulative Incidence of Death From Any Cause at 1 Year in Patients With an Adjudicated Diagnosis of Type 1 Myocardial Infarction and Adjudicated Diagnosis of Type 2 Myocardial Infarction Stratified According to Those With a Clinical Diagnosis and Without a Clinical Diagnosis of Myocardial Infarction **eFigure 5.** Cumulative Incidence of Death From Cardiovascular Causes at 1 Year in Patients With an Adjudicated Diagnosis of Type 1 Myocardial Infarction and Adjudicated Diagnosis of Type 2 Myocardial Infarction Stratified According to Those With a Clinical Diagnosis and Without a Clinical Diagnosis of Myocardial Infarction **eFigure 6.** Cumulative Incidence Death From Noncardiovascular Causes at 1 Year in Patients With an Adjudicated Diagnosis of Type 1 Myocardial Infarction and Adjudicated Diagnosis of Type 2 Myocardial Infarction Stratified According to Those With a Clinical Diagnosis and Without a Clinical Diagnosis of Myocardial Infarction
eReferences.
**eTable 1.** Clinical Characteristics of Cohorts From Scotland and Sweden **eTable 2.** Characteristics Associated With a Clinical Diagnosis of Myocardial Infarction Stratified by the Universal Definition **eTable 3.** Management and Outcomes of Patients With and Without a Clinical Diagnosis of Myocardial Infarction Stratified by the Universal Definition **eTable 4.** Risk of Subsequent Myocardial Infarction or Cardiovascular Death at 1 Year in Patients With a Clinical Diagnosis of Myocardial Infarction Compared to Those Without a Clinical Diagnosis Stratified by the Universal Definition **eTable 5.** Clinical Diagnosis of Myocardial Infarction Using Different Positions in the Hospital Record for Patients With an Adjudicated Diagnosis in Scotland **eTable 6.** Additional Analysis Stratifying by the Presence of ST-Elevation on the Electrocardiogram and Effect on the Diagnostic Performance of a Clinical Diagnosis of Myocardial Infarction in the Hospital Record Stratified by the Universal Definition **eTable 7.** Characteristics Associated With a Clinical Diagnosis in Populations of Both ST Segment Elevation and Non-ST-Elevation Myocardial Infarctions in Scotland

Supplement 2.
Data Sharing Statement

## References

[1] Thygesen K, Alpert JS, Jaffe AS, ; ESC Scientific Document Group. Fourth universal definition of myocardial infarction (2018). Eur Heart J. 2019;40(3):237-269. doi:10.1093/eurheartj/ehy462 30165617

[2] Collet JP, Thiele H, Barbato E, ; ESC Scientific Document Group. 2020 ESC Guidelines for the management of acute coronary syndromes in patients presenting without persistent ST-segment elevation. Eur Heart J. 2021;42(14):1289-1367. doi:10.1093/eurheartj/ehaa575 32860058

[3] Gulati M, Levy PD, Mukherjee D, ; Writing Committee Members. 2021 AHA/ACC/ASE/CHEST/SAEM/SCCT/SCMR guideline for the evaluation and diagnosis of chest pain: a report of the American College of Cardiology/American Heart Association Joint Committee on Clinical Practice Guidelines. J Am Coll Cardiol. 2021;78(22):e187-e285. doi:10.1016/j.jacc.2021.07.053 34756653

[4] Thygesen K, Alpert JS, White HD, ; Joint ESC/ACCF/AHA/WHF Task Force for the Redefinition of Myocardial Infarction. Universal definition of myocardial infarction. Circulation. 2007;116(22):2634-2653. doi:10.1161/CIRCULATIONAHA.107.187397 17951284

[5] Alpert JS, Thygesen KA, White HD, Jaffe AS. Diagnostic and therapeutic implications of type 2 myocardial infarction: review and commentary. Am J Med. 2014;127(2):105-108. doi:10.1016/j.amjmed.2013.09.031 24462011

[6] Collinson P, Lindahl B. Type 2 myocardial infarction: the chimaera of cardiology? Heart. 2015;101(21):1697-1703. doi:10.1136/heartjnl-2014-307122 26220812

[7] Chapman AR, Sandoval Y. Type 2 myocardial infarction: evolving approaches to diagnosis and risk-stratification. Clin Chem. 2021;67(1):61-69. doi:10.1093/clinchem/hvaa189 33418588 PMC7793229

[8] Sandoval Y, Jaffe AS. Type 2 myocardial infarction: JACC review topic of the week. J Am Coll Cardiol. 2019;73(14):1846-1860. doi:10.1016/j.jacc.2019.02.018 30975302

[9] DeFilippis AP, Chapman AR, Mills NL, . Assessment and treatment of patients with type 2 myocardial infarction and acute nonischemic myocardial injury. Circulation. 2019;140(20):1661-1678. doi:10.1161/CIRCULATIONAHA.119.040631 31416350 PMC6855329

[10] Chapman AR, Shah ASV, Lee KK, . Long-term outcomes in patients with type 2 myocardial infarction and myocardial injury. Circulation. 2018;137(12):1236-1245. doi:10.1161/CIRCULATIONAHA.117.031806 29150426 PMC5882250

[11] Chapman AR, Adamson PD, Shah ASV, ; High-STEACS Investigators. High-sensitivity cardiac troponin and the universal definition of myocardial infarction. Circulation. 2020;141(3):161-171. doi:10.1161/CIRCULATIONAHA.119.042960 31587565 PMC6970546

[12] Raphael CE, Roger VL, Sandoval Y, . Incidence, trends, and outcomes of type 2 myocardial infarction in a community cohort. Circulation. 2020;141(6):454-463. doi:10.1161/CIRCULATIONAHA.119.043100 31902228 PMC8283933

[13] McCarthy CP, Kolte D, Kennedy KF, Vaduganathan M, Wasfy JH, Januzzi JL Jr. Patient characteristics and clinical outcomes of type 1 versus type 2 myocardial infarction. J Am Coll Cardiol. 2021;77(7):848-857. doi:10.1016/j.jacc.2020.12.034 33602466

[14] Tripathi B, Tan BEX, Sharma P, . Characteristics and outcomes of patients admitted with type 2 myocardial infarction. Am J Cardiol. 2021;157:33-41. doi:10.1016/j.amjcard.2021.07.013 34373076

[15] Bularga A, Taggart C, Mendusic F, ; High-Sensitivity Troponin in the Evaluation of Patients with Suspected Acute Coronary Syndrome (High-STEACS) Investigators. Assessment of oxygen supply-demand imbalance and outcomes among patients with type 2 myocardial infarction: a secondary analysis of the High-STEACS cluster randomized clinical trial. JAMA Netw Open. 2022;5(7):e2220162. doi:10.1001/jamanetworkopen.2022.20162 35816305 PMC9274319

[16] Scottish Government. Charter for Safe Havens in Scotland: handling unconsented data from national health service patient records to support research and statistics. Accessed March 8, 2024. https://www.gov.scot/publications/charter-safe-havens-scotland-handling-unconsented-data-national-health-service-patient-records-support-research-statistics/pages/1/

[17] Swedish Code of Statutes. Act (2003:460) on the Ethical Review of Research Involving Humans. Accessed March 8, 2024. https://www.riksdagen.se/sv/dokument-och-lagar/dokument/svensk-forfattningssamling/lag-2003460-om-etikprovning-av-forskning-som_sfs-2003-460/

[18] Shah ASV, Anand A, Strachan FE, ; High-STEACS Investigators. High-sensitivity troponin in the evaluation of patients with suspected acute coronary syndrome: a stepped-wedge, cluster-randomised controlled trial. Lancet. 2018;392(10151):919-928. doi:10.1016/S0140-6736(18)31923-8 30170853 PMC6137538

[19] Kadesjö E, Roos A, Siddiqui A, Desta L, Lundbäck M, Holzmann MJ. Acute versus chronic myocardial injury and long-term outcomes. Heart. 2019;105(24):1905-1912. doi:10.1136/heartjnl-2019-315036 31337668

[20] Shah ASV, Griffiths M, Lee KK, . High sensitivity cardiac troponin and the under-diagnosis of myocardial infarction in women: prospective cohort study. BMJ. Published online January 21, 2015. doi:10.1136/bmj.g7873 25609052 PMC4301191

[21] Giannitsis E, Kurz K, Hallermayer K, Jarausch J, Jaffe AS, Katus HA. Analytical validation of a high-sensitivity cardiac troponin T assay. Clin Chem. 2010;56(2):254-261. doi:10.1373/clinchem.2009.132654 19959623

[22] Gard A, Lindahl B, Batra G, Hjort M, Szummer K, Baron T. Diagnosing type 2 myocardial infarction in clinical routine: a validation study. Scand Cardiovasc J. 2019;53(5):259-265. doi:10.1080/14017431.2019.1638961 31294615

[23] Bularga A, Hung J, Daghem M, . Coronary artery and cardiac disease in patients with type 2 myocardial infarction: a prospective cohort study. Circulation. 2022;145(16):1188-1200. doi:10.1161/CIRCULATIONAHA.121.058542 35341327 PMC9010024

[24] Wereski R, Kimenai DM, Bularga A, . Risk factors for type 1 and type 2 myocardial infarction. Eur Heart J. 2022;43(2):127-135. doi:10.1093/eurheartj/ehab581 34431993 PMC8757580

[25] Lindahl B, Mills NL. A new clinical classification of acute myocardial infarction. Nat Med. 2023;29(9):2200-2205. doi:10.1038/s41591-023-02513-2 37635156

[26] Braunwald E. Unstable angina: a classification. Circulation. 1989;80(2):410-414. doi:10.1161/01.CIR.80.2.410 2752565

[27] van der Ende MY, Juarez-Orozco LE, Waardenburg I, . Sex-based differences in unrecognized myocardial infarction. J Am Heart Assoc. 2020;9(13):e015519. doi:10.1161/JAHA.119.015519 32573316 PMC7670510

[28] de Torbal A, Boersma E, Kors JA, . Incidence of recognized and unrecognized myocardial infarction in men and women aged 55 and older: the Rotterdam Study. Eur Heart J. 2006;27(6):729-736. doi:10.1093/eurheartj/ehi707 16478749

[29] Rashid S, Simms A, Batin P, Kurian J, Gale CP. Inequalities in care in patients with acute myocardial infarction. World J Cardiol. 2015;7(12):895-901. doi:10.4330/wjc.v7.i12.895 26730295 PMC4691816

[30] World Health Organization. International Statistical Classification of Diseases, Tenth Revision (ICD-10). World Health Organization; 1992.

[31] Goyal A, Gluckman TJ, Tcheng JE. What’s in a Name: the new *ICD-10* (*10th Revision of the International Statistical Classification of Diseases and Related Health Problems*) codes and type 2 myocardial infarction. Circulation. 2017;136(13):1180-1182. doi:10.1161/CIRCULATIONAHA.117.030347 28947477

[32] McCarthy C, Murphy S, Cohen JA, . Misclassification of myocardial injury as myocardial infarction: implications for assessing outcomes in value-based programs. JAMA Cardiol. 2019;4(5):460-464. doi:10.1001/jamacardio.2019.0716 30879022 PMC6537800

