# Supplementary material for: Application of the Universal Definition of Myocardial Infarction in Clinical Practice in Scotland and Sweden
Source: JAMA Netw Open. 2024 Apr 8;7(4):e245853. doi: 10.1001/jamanetworkopen.2024.5853 (PMC11002705; doi:10.1001/jamanetworkopen.2024.5853)
Supplement: Supplement 1. — eMethods. Adjudication Process eFigure 1. Proportion of Patients With an Adjudicated Diagnosis of Type 1 Myocardial Infarction and Type 2 Myocardial Infarction Who Had a Clinical Diagnosis of Myocardial Infarction in the Hospital Record Over 6-Month Blocks in Scotland and Sweden eFigure 2. Cumulative Incidence of Cardiovascular Death or Myocardial Infarction in All Universal Definition Subtypes of Myocardial Infarction With No ST Elevation and ST Elevations Stratified According to Those With a Clinical Diagnosis and Without a Clinical Diagnosis of Myocardial Infarction eFigure 3. Cumulative Incidence of All Cause Death in All Myocardial Infarctions in Scotland and Sweden Stratified According to Those With a Clinical Diagnosis and Without a Clinical Diagnosis of Myocardial Infarction eFigure 4. Cumulative Incidence of Death From Any Cause at 1 Year in Patients With an Adjudicated Diagnosis of Type 1 Myocardial Infarction and Adjudicated Diagnosis of Type 2 Myocardial Infarction Stratified According to Those With a Clinical Diagnosis and Without a Clinical Diagnosis of Myocardial Infarction eFigure 5. Cumulative Incidence of Death From Cardiovascular Causes at 1 Year in Patients With an Adjudicated Diagnosis of Type 1 Myocardial Infarction and Adjudicated Diagnosis of Type 2 Myocardial Infarction Stratified According to Those With a Clinical Diagnosis and Without a Clinical Diagnosis of Myocardial Infarction eFigure 6. Cumulative Incidence Death From Noncardiovascular Causes at 1 Year in Patients With an Adjudicated Diagnosis of Type 1 Myocardial Infarction and Adjudicated Diagnosis of Type 2 Myocardial Infarction Stratified According to Those With a Clinical Diagnosis and Without a Clinical Diagnosis of Myocardial Infarction eReferences. eTable 1. Clinical Characteristics of Cohorts From Scotland and Sweden eTable 2. Characteristics Associated With a Clinical Diagnosis of Myocardial Infarction Stratified by the Universal Definition eTable 3. Management and Outcomes o [file jamanetwopen-e245853-s001.pdf]

Supplemental Online Content

Taggart C, Roos A, Kadesjö E, et al. Application of the universal definition of myocardial infarction in clinical practice in Scotland and Sweden. *JAMA Netw Open*. 2024;7(4):e245853. doi:10.1001/jamanetworkopen.2024.5853

eMethods. Adjudication Process
eFigure 1. Proportion of Patients With an Adjudicated Diagnosis of Type 1 Myocardial Infarction and Type 2 Myocardial Infarction Who Had a Clinical Diagnosis of Myocardial Infarction in the Hospital Record Over 6-Month Blocks in Scotland and Sweden
eFigure 2. Cumulative Incidence of Cardiovascular Death or Myocardial Infarction in All Universal Definition Subtypes of Myocardial Infarction With No ST Elevation and ST Elevations Stratified According to Those With a Clinical Diagnosis and Without a Clinical Diagnosis of Myocardial Infarction
eFigure 3. Cumulative Incidence of All Cause Death in All Myocardial Infarctions in Scotland and Sweden Stratified According to Those With a Clinical Diagnosis and Without a Clinical Diagnosis of Myocardial Infarction
eFigure 4. Cumulative Incidence of Death From Any Cause at 1 Year in Patients With an Adjudicated Diagnosis of Type 1 Myocardial Infarction and Adjudicated Diagnosis of Type 2 Myocardial Infarction Stratified According to Those With a Clinical Diagnosis and Without a Clinical Diagnosis of Myocardial Infarction
eFigure 5. Cumulative Incidence of Death From Cardiovascular Causes at 1 Year in Patients With an Adjudicated Diagnosis of Type 1 Myocardial Infarction and Adjudicated Diagnosis of Type 2 Myocardial Infarction Stratified According to Those With a Clinical Diagnosis and Without a Clinical Diagnosis of Myocardial Infarction
eFigure 6. Cumulative Incidence Death From Noncardiovascular Causes at 1 Year in Patients With an Adjudicated Diagnosis of Type 1 Myocardial Infarction and Adjudicated Diagnosis of Type 2 Myocardial Infarction Stratified According to Those With a Clinical Diagnosis and Without a Clinical Diagnosis of Myocardial Infarction
eReferences.
eTable 1. Clinical Characteristics of Cohorts From Scotland and Sweden
eTable 2. Characteristics Associated With a Clinical Diagnosis of Myocardial Infarction Stratified by the Universal Definition
eTable 3. Management and Outcomes of Patients With and Without a Clinical Diagnosis of Myocardial Infarction Stratified by the Universal Definition
eTable 4. Risk of Subsequent Myocardial Infarction or Cardiovascular Death at 1 Year in Patients With a Clinical Diagnosis of Myocardial Infarction Compared to Those Without a Clinical Diagnosis Stratified by the Universal Definition
eTable 5. Clinical Diagnosis of Myocardial Infarction Using Different Positions in the Hospital Record for Patients With an Adjudicated Diagnosis in Scotland
eTable 6. Additional Analysis Stratifying by the Presence of ST-Elevation on the Electrocardiogram and Effect on the Diagnostic Performance of a Clinical Diagnosis of Myocardial Infarction in the Hospital Record Stratified by the Universal Definition
eTable 7. Characteristics Associated With a Clinical Diagnosis in Populations of Both ST Segment Elevation and Non-ST-Elevation Myocardial Infarctions in Scotland

This supplemental material has been provided by the authors to give readers additional information about their work.

eMethods. Adjudication process

Study approvals

The High-STEACS trial was approved by the Scotland A Research Ethics Committee, the Public Benefit and Privacy Panel for Health and Social Care, and by each National Health Service Health Board. Individual patient consent was not required and data from consecutive patients was collected prospectively from the electronic record, deidentified and linked within a Secure Data Environment in the National Health Service (Dataloch, Edinburgh, United Kingdom). For the cohort in Sweden, the study protocol was approved by the Regional Ethical Review Board in Stockholm. Both studies were conducted in accordance with the Declaration of Helsinki. Inc0681807

Patient and public involvement

Patients and lay representatives were members of the steering committee for the trial and all related studies and were involved in the design, conduct and approval of the High-STEACS trial.

Adjudication processes

All patients with high-sensitivity cardiac troponin I concentrations above the sex-specific 99th centile were classified according to the Third Universal Definition of Myocardial Infarction in use at the time the trial was conducted. For this secondary analysis, we updated this classification in accordance with the Fourth Universal Definition of Myocardial Infarction. Two physicians independently reviewed all clinical information, blinded to study phase, with discordant diagnoses resolved by a third reviewer. Adjudication was performed by 11 different reviewers using a bespoke electronic console that combined relevant deidentified

© 2024 Taggart C et al. *JAMA Network Open.*

clinical information from multiple sources, including the electronic clinical record. The adjudicators were all experienced clinicians with training in both general medicine and cardiology.

Type 1 myocardial infarction was defined as myocardial necrosis (any cardiac troponin I concentration above the sex-specific 99th centile with a rise and/or fall concentration where serial testing was performed) in the context of a presentation with suspected acute coronary syndrome with symptoms or signs of myocardial ischemia on the electrocardiogram. Patients with symptoms or signs of myocardial ischemia and evidence of increased oxygen demand or decreased supply (for example, tachyarrhythmia, hypotension, or anemia) secondary to an alternative pathology and myocardial necrosis were defined as type 2 myocardial infarction. The classification of type 2 myocardial infarction also includes patients with coronary mechanisms including vasospasm, embolism, or spontaneous dissection without evidence of atherothrombosis. Type 4a myocardial infarction was defined in patients with symptoms or signs of myocardial ischemia following percutaneous coronary intervention where cardiac troponin I concentrations were 5-fold greater than the sex-specific 99th centile or increased further if elevated prior to the procedure. Type 4b myocardial infarction was defined where myocardial ischemia and myocardial necrosis were associated with stent thrombosis documented at angiography. Myocardial injury was defined if cardiac troponin I concentrations were above the sex-specific 99th centile in the absence of any clinical features of myocardial ischemia.

A similar adjudication process was applied in the cohort from Sweden through direct review of the clinical record by three experienced reviewers with training in emergency medicine and cardiology. Myocardial infarction was defined, and cardiac troponin concentrations interpreted as recommended by current guidelines [1,2]. In brief, myocardial infarction was

© 2024 Taggart C et al. *JAMA Network Open.*

diagnosed when there was evidence of myocardial necrosis with a significant rise and/or fall in a clinical setting consistent with myocardial ischemia. Patients with myocardial infarction were stratified into those with type 1 (primary coronary events) and type 2 (ischemia due to increased demand or decreased supply, for example tachyarrhythmia or hypertensive crisis) myocardial infarction [1,3]. All other patients were classified in the categories of acute non-ischemic myocardial injury or chronic myocardial injury. All non-ischaemic myocardial injury in both cohorts was classified as acute, unless a change of <20% was observed on serial testing.In the cohort from Scotland, there was good agreement between clinical adjudicators (Cohen's Kappa κ) = 0.75. Agreement was higher in patients with type 1 myocardial infarction (κ = 0.78) and myocardial injury (κ = 0.65) as compared with type 2 myocardial infarction (κ = 0.49) [4]. In the Swedish cohort the interobserver variability for 78 randomly chosen cases were 77%, 85% and 81% for three adjudicators. The intra-observer variability was 96% for all adjudicators [5].

© 2024 Taggart C et al. *JAMA Network Open.*

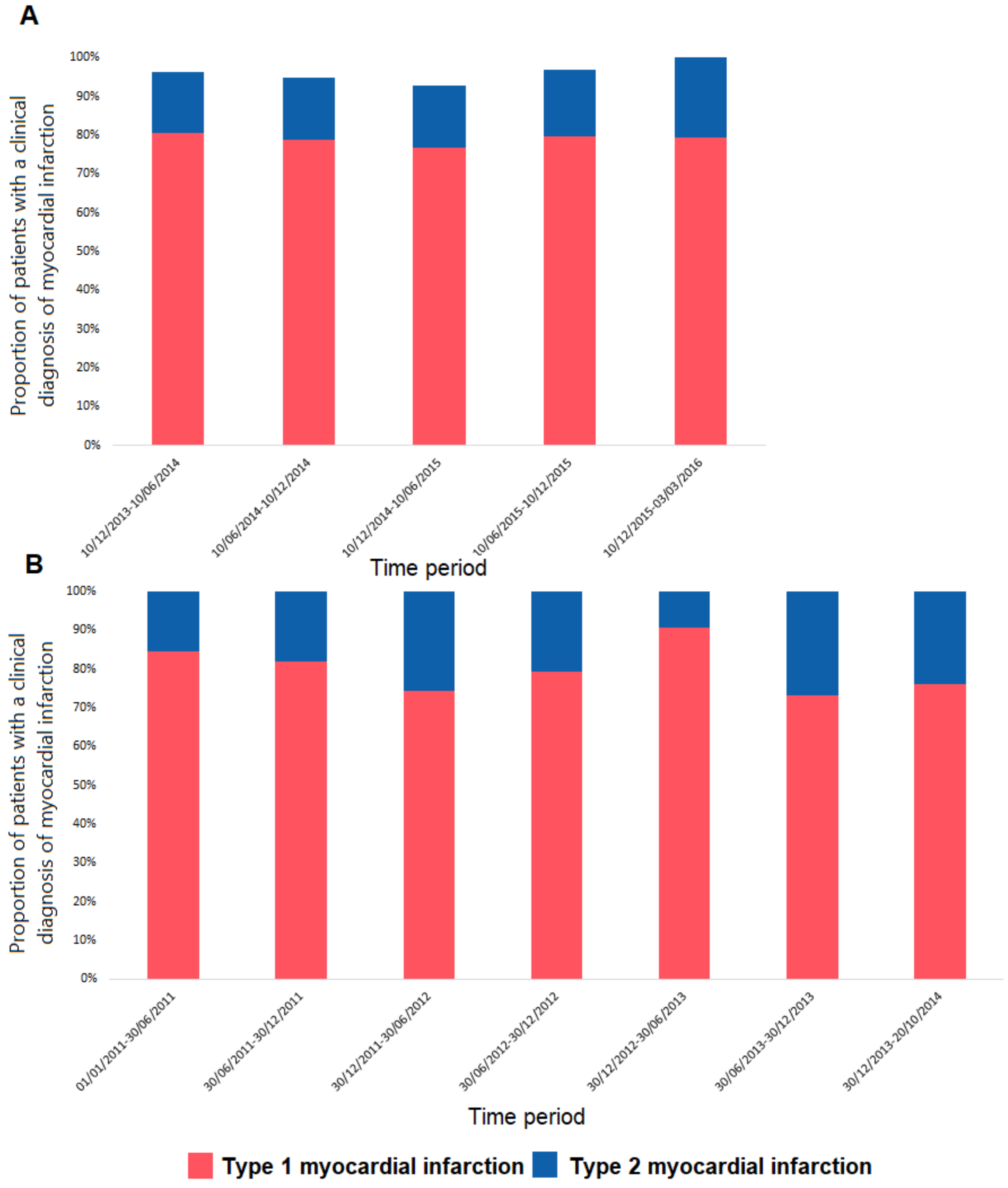

eFigure 1. Proportion of patients with an adjudicated diagnosis of type 1 myocardial infarction (red) and type 2 myocardial infarction (blue) who had a clinical diagnosis of myocardial infarction in the hospital record over 6-month blocks in Scotland (panel A) and Sweden (panel B).

© 2024 Taggart C et al. *JAMA Network Open.*

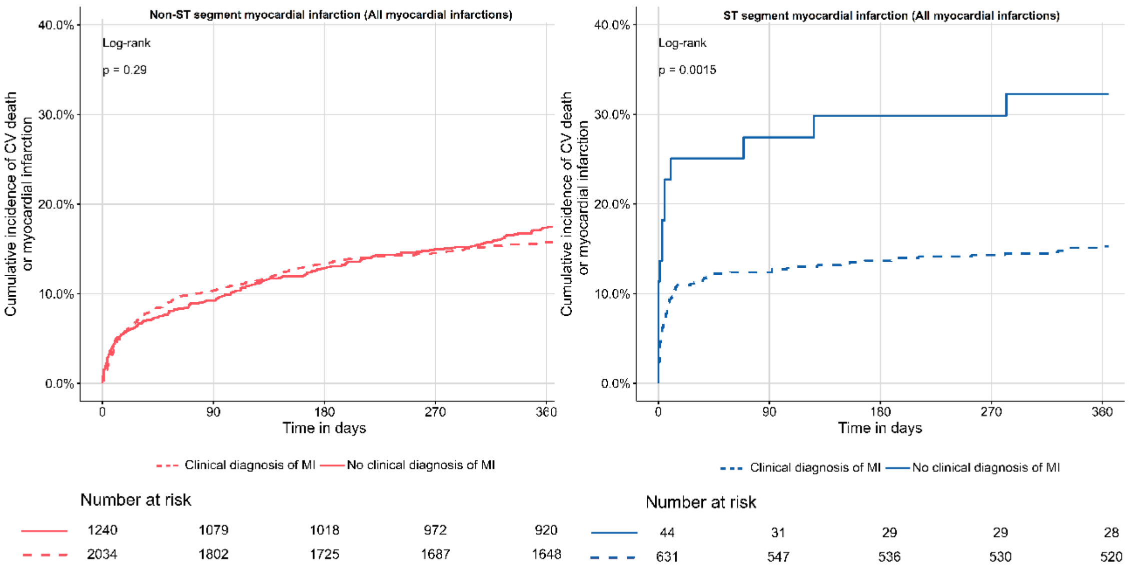

eFigure 2. Cumulative incidence of Cardiovascular death or myocardial infarction in all universal definition subtypes of myocardial infarction with no ST elevation (left panel) and ST elevations (right panel) stratified according to those with a clinical diagnosis (dashed line) and without a clinical diagnosis of myocardial infarction (solid line).

© 2024 Taggart C et al. *JAMA Network Open.*

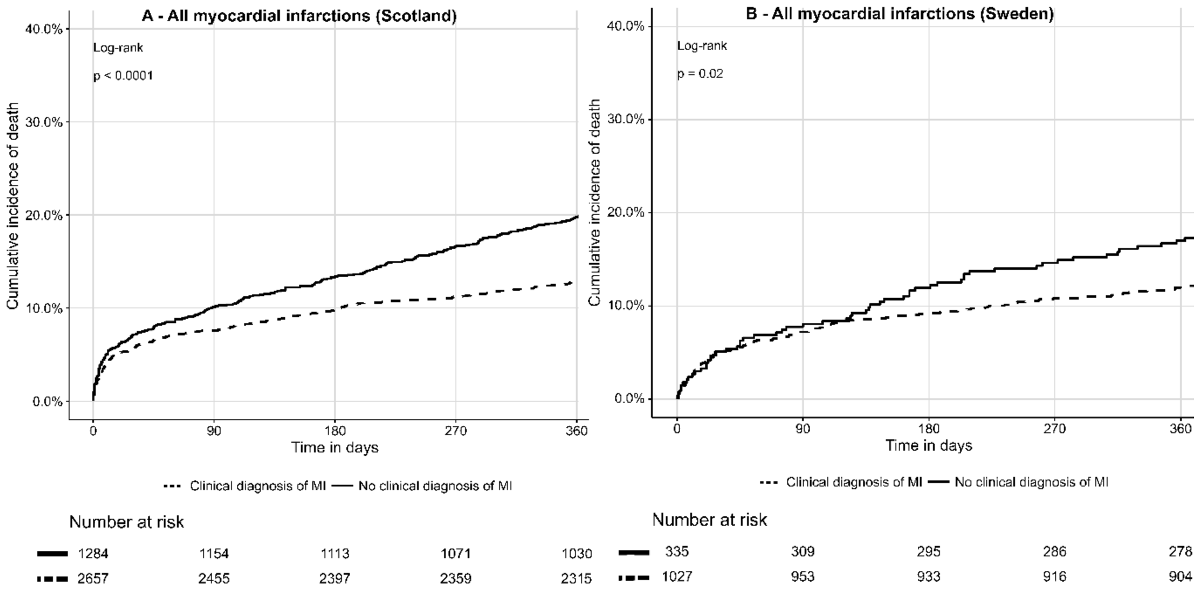

eFigure 3. Cumulative incidence of all cause death in all myocardial infarctions in Scotland (panel A) and Sweden (panel B) stratified according to those with a clinical diagnosis (dashed line) and without a clinical diagnosis of myocardial infarction (solid line).

© 2024 Taggart C et al. *JAMA Network Open.*

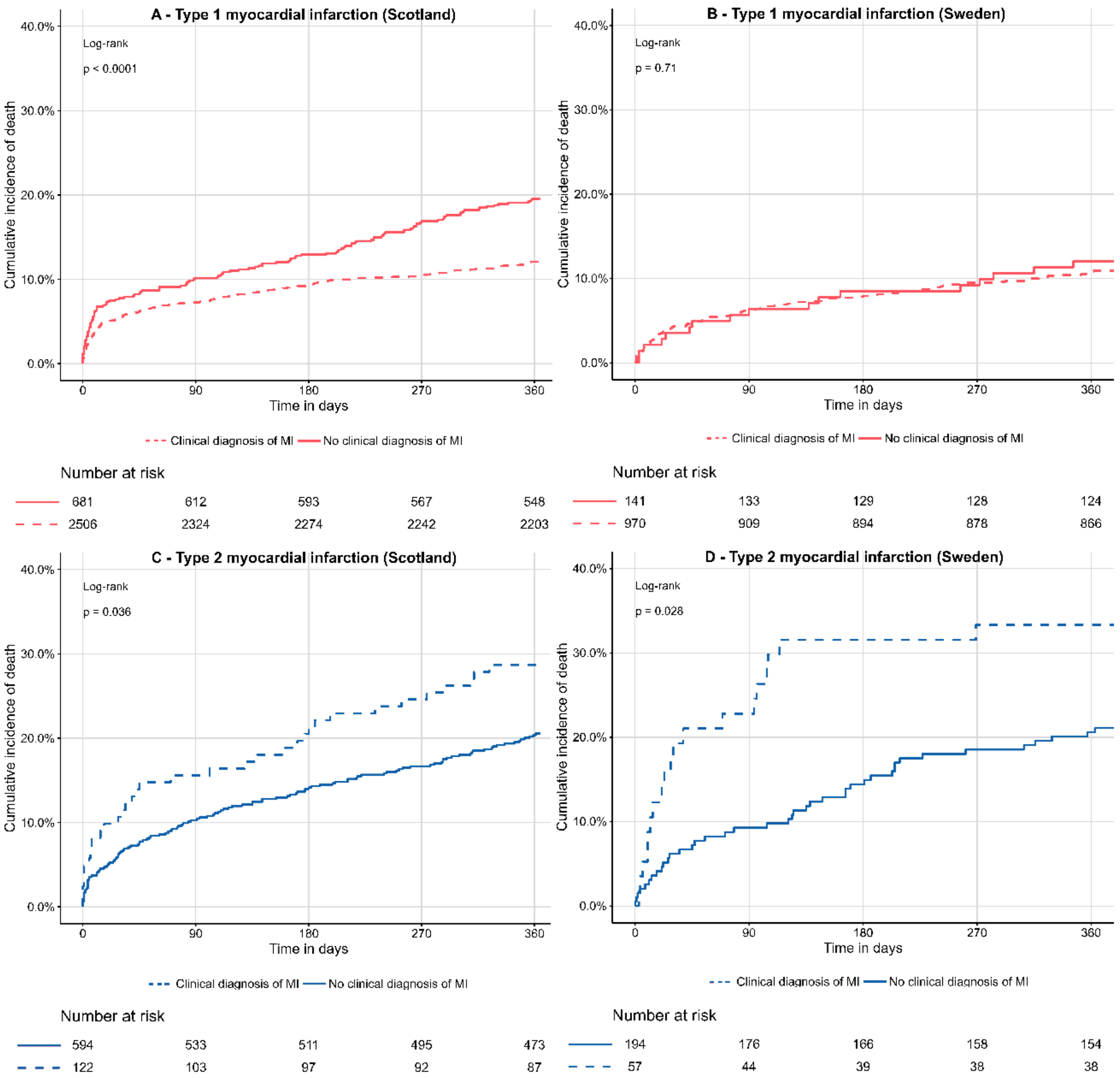

eFigure 4. Cumulative incidence of death from any cause at 1 year in patients with an adjudicated diagnosis of type 1 myocardial infarction (panel A-B) and adjudicated diagnosis of type 2 myocardial infarction (panel C-D) stratified according to those with a clinical diagnosis (dashed line) and without a clinical diagnosis of myocardial infarction (solid line).

© 2024 Taggart C et al. *JAMA Network Open.*

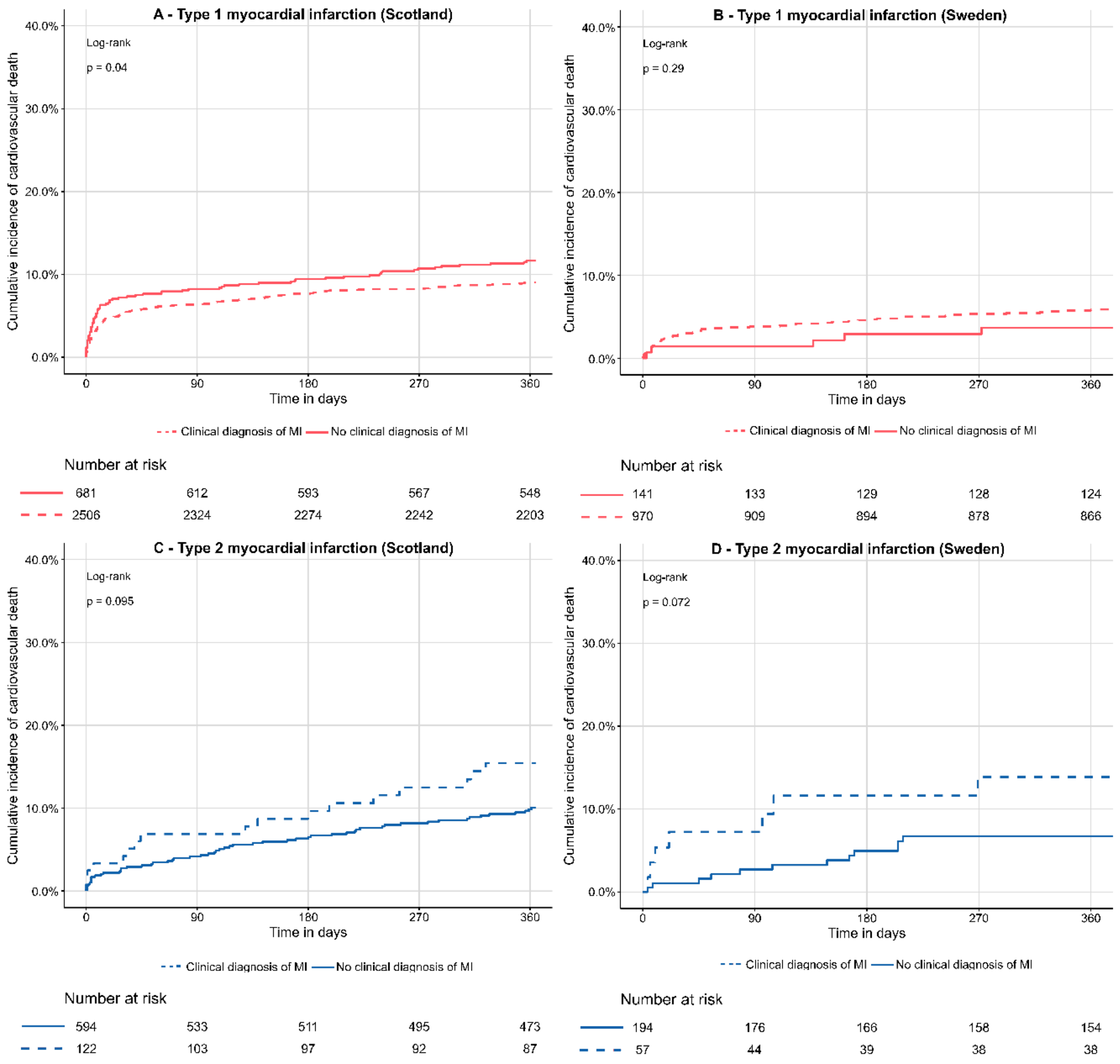

eFigure 5. Cumulative incidence of death from cardiovascular causes at 1 year in patients with an adjudicated diagnosis of type 1 myocardial infarction (panel A-B) and adjudicated diagnosis of type 2 myocardial infarction (panel C-D) stratified according to those with a clinical diagnosis (dashed line) and without a clinical diagnosis of myocardial infarction (solid line).

© 2024 Taggart C et al. *JAMA Network Open.*

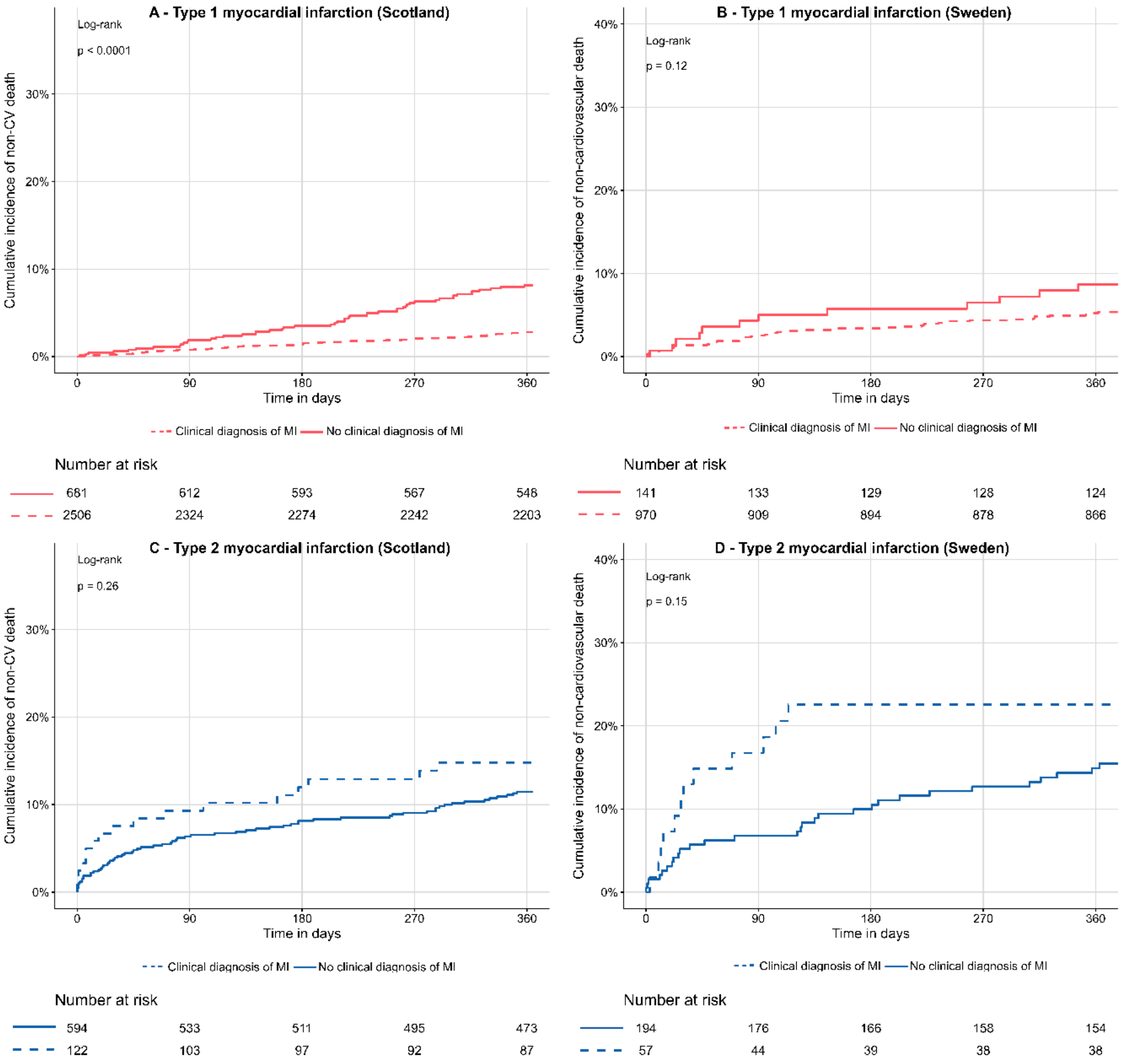

eFigure 6. Cumulative incidence death from non-cardiovascular causes at 1 year in patients with an adjudicated diagnosis of type 1 myocardial infarction (panel A-B) and adjudicated diagnosis of type 2 myocardial infarction (panel C-D) stratified according to those with a clinical diagnosis (dashed line) and without a clinical diagnosis of myocardial infarction (solid line).

© 2024 Taggart C et al. *JAMA Network Open.*

eReferences

1. Thygesen K, Alpert JS, Jaffe AS, et al. Fourth universal definition of myocardial infarction (2018). *Eur Heart J*. 2018;40:237-269.
2. Thygesen K, Mair J, Giannitsis E, et al. How to use high-sensitivity cardiac troponins in acute cardiac care. *Eur Heart J*. 2012;33(18):2252-2257. doi:10.1093/eurheartj/ehs154
3. Collet J-P, Thiele H, Barbato E, et al. 2020 ESC Guidelines for the management of acute coronary syndromes in patients presenting without persistent ST-segment elevation: The Task Force for the management of acute coronary syndromes in patients presenting without persistent ST-segment elevation. *Eur Heart J*. Published online August 2020:ehaa575. doi:10.1093/eurheartj/ehaa575
4. Chapman AR, Adamson PD, Shah ASV, et al. High-sensitivity cardiac troponin and the universal definition of myocardial infarction. *Circulation*. 2020;141:161-171. doi:10.1161/CIRCULATIONAHA.119.042960
5. Kadesjö E, Roos A, Siddiqui A, Desta L, Lundbäck M, Holzmann MJ. Acute versus chronic myocardial injury and long-term outcomes. *Heart*. 2019;105(24):1905-1912. doi:10.1136/heartjnl-2019-315036

© 2024 Taggart C et al. *JAMA Network Open.*

eTable 1. Clinical characteristics of cohorts from Scotland and Sweden

	Cohort from Scotland		Cohort from Sweden	
	**All**<br>n = 28,783	**Any myocardial infarction**<br>n = 3,941	**All**<br>n = 21,573	**Any myocardial infarction**<br>n = 1,362
Age (years)	60 (17)	69 (14)	56 (17)	69 (13)
Sex (males)	15,562 (54)	2,303 (58)	11,110 (51)	882 (65)
Chest pain at presentation (yes)	23,584 (82)	3,390 (86)	21,573 (100)	1,362 (100)
Past medical history (yes)				
Myocardial infarction	2,185 (7.6)	487 (12)	1,606 (7.4)	240 (18)
Ischemic heart disease	6,350 (22)	1,171 (30)	2,264 (10)	345 (25)
Cerebrovascular disease	1,552 (5.4)	317 (8.0)	807 (3.7)	90 (6.6)
Diabetes Mellitus	1,886 (6.6)	596 (15)	2,012 (9.3)	269 (20)
HF hospitalization	2,230 (7.7)	605 (15)	189 (14)[a]	189 (14)
Revascularisation	2,810 (8.7)	459 (11.7)	1,807 (8.4)	270 (20)
Electrocardiogram				
Myocardial ischemia	1,706 (34)[b]	1,584 (44)	367 (27)[a]	367 (27)
ST segment elevation	732 (15)	674 (19)	#	#
ST depression	855 (17)	772 (22)	#	#
T wave inversion	773 (16)	611 (17)	#	#
Heart rate (bpm)	85 (26)	83 (25)	80 (23)	79 (21)
SBP (mmHg)	139 (29)	140 (29)	148 (29)	151 (29)
Hemoglobin (g/L)	138 (18)	136 (21)	133 (19)	137 (20)
eGFR (mL/min)	69 (27)	73 (26)	89 (23)	73 (24)
Peak troponin I (ng/l)	4 (1, 14)	667 (93, 5,914)	#	#
Peak troponin T (ng/l)	#	#	5 (5, 10)	143 (44, 514)

Continuous variables are presented as mean (SD) or median (25th, 75th percentile), as appropriate. Categorical variables are presented as number (%). Missing values were under <5% if applicable in both cohorts [a]Electrocardiogram data and heart failure hospitalization was only available in patients with an adjudicated diagnosis of myocardial infarction in the Swedish dataset. [b]Only patients with troponin >99th centile had electrocardiogram data in the Scottish dataset.

© 2024 Taggart C et al. *JAMA Network Open.*

eTable 2. Characteristics associated with a clinical diagnosis of myocardial infarction stratified by the universal definition

	Odds ratio of a clinical diagnosis of myocardial infarction in Scotland		Odds ratio of a clinical diagnosis of myocardial infarction in Sweden	
	**Univariable model OR (95% CI)**	**Multivariable model OR (95% CI)**	**Univariable model OR (95% CI)**	**Multivariable model OR (95% CI)**
**Adjudicated diagnosis of type 1 myocardial infarction**				
Age (per 10 years)	0.78 (0.73 to 0.83)	0.96 (0.88 to 1.04)	0.91 (0.80 to 1.06)	1.15 (0.95 to 1.40)
Sex (women)	0.58 (0.49 to 0.69)	1.06 (0.87 to 1.31)	0.71 (0.50 to 1.03)	0.89 (0.58 to 1.35)
Ischemic heart disease (yes)	0.44 (0.37 to 0.52)	**0.75 (0.60 to 0.93)**	0.60 (0.41 to 0.87)	0.71 (0.47 to 1.08)
Cerebrovascular disease (yes)	0.49 (0.37 to 0.65)	0.73 (0.52 to 1.02)	1.00 (0.49 to 2.06)	1.05 (0.48 to 2.30)
Diabetes mellitus (yes)	1.00 (0.80 to 1.27)	**1.56 (1.18 to 2.06)**	0.82 (0.53 to 1.26)	1.05 (0.63 to 1.62)
Heart failure hospitalization (yes)	0.52 (0.42 to 0.65)	0.78 (0.59 to 1.03)	0.63 (0.39 to 1.02)	0.68 (0.40 to 1.20)
ECG ischemia (yes)	2.89 (2.39 to 3.49)	**1.72 (1.39 to 2.12)**	1.10 (0.73 to 1.64)	0.82 (0.53 to 1.28)
eGFR (per 10 ml/min)	1.16 (1.12 to 1.20)	**1.13 (1.08 to 1.18)**	1.13 (1.05 to 1.22)	**1.18 (1.06 to 1.30)**
Hemoglobin (per 10 mg/l)	1.20 (1.15 to 1.25)	1.05 (0.99 to 1.11)	1.11 (1.00 to 1.23)	1.04 (0.92 to 1.19)
Cardiac troponin (per log)	1.36 (1.32 to 1.40)	**1.32 (1.28 to 1.37)**	1.55 (1.40 to 1.72)	**1.61 (1.45 to 1.79)**
**Adjudicated diagnosis of type 2 myocardial infarction**				
Age (per 10 years)	1.04 (0.90 to 1.20)	1.08 (0.89 to 1.31)	1.31 (1.04 to 1.67)	1.26 (0.93 to 1.71)
Sex (women)	0.93 (0.63 to 1.38)	1.52 (0.95 to 2.44)	0.76 (0.42 to 1.37)	0.85 (0.44 to 1.66)
Ischemic heart disease (yes)	1.49 (1.00 to 2.21)	**1.98 (1.16 to 3.36)**	1.07 (0.55 to 2.06)	0.95 (0.45 to 1.99)
Cerebrovascular disease (yes)	1.61 (0.92 to 2.81)	1.79 (0.89 to 3.57)	1.24 (0.42 to 3.59)	1.15 (0.35 to 3.80)
Diabetes mellitus (yes)	1.43 (0.82 to 2.47)	0.90 (0.43 to 1.87)	1.18 (0.59 to 2.36)	0.80 (0.36 to 1.80)
Heart failure hospitalization (yes)	1.33 (0.86 to 2.06)	0.92 (0.54 to 1.59)	1.60 (0.81 to 3.16)	1.49 (0.70 to 3.18)
ECG ischemia (yes)	1.79 (1.21 to 2.65)	1.32 (0.84 to 2.09)	1.40 (0.74 to 2.68)	0.95 (0.46 to 1.95)
eGFR (per 10 ml/min)	1.00 (0.93 to 1.08)	**1.20 (1.08 to 1.34)**	0.95 (0.84 to 1.07)	1.11 (0.95 to 1.30)
Hemoglobin (per 10 mg/l)	0.87 (0.80 to 0.93)	0.89 (0.81 to 0.98)	0.91 (0.82 to 1.01)	0.95 (0.84 to 1.07)
Cardiac troponin (per log)	1.54 (1.42 to 1.68)	**1.61 (1.47 to 1.76)**	1.57 (1.31 to 1.90)	**1.58 (1.29 to 1.93)**

Abbreviations: ECG, electrocardiogram; eGFR, estimated Glomerular Filtration rate; MI, myocardial infarction.

© 2024 Taggart C et al. *JAMA Network Open.*

eTable 3. Management and outcomes of patients with and without a clinical diagnosis of myocardial infarction stratified by the universal definition

	**Clinical diagnosis of myocardial infarction in Scotland**		**Clinical diagnosis of myocardial infarction in Sweden**	
	Yes (n = 2,506)	No (n = 681)	Yes (n = 970)	No (n = 141)
**Adjudicated diagnosis of type 1 myocardial infarction**				
Coronary angiogram at 30 days	1,883 (75)	176 (26)	704 (73)	77 (55)
Revascularisation at 30 days	1,431 (57)	94 (14)	524 (54)	53 (38)
Aspirin at 30 days	2,104 (84)	401 (59)	694 (72)	75 (53)
Antiplatelet therapy at 30 days	2,263 (90)	482 (71)	862 (89)	94 (67)
Statin at 30 days	2,180 (87)	488 (72)	692 (71)	78 (55)
Betablocker at 30 days	1,806 (72)	383 (56)	715 (74)	88 (62)
ACE inhibitor at 30 days	1,877 (75)	386 (57)	496 (51)	57 (40)
Cardiovascular death or MI at one year	386 (15)	145 (21)	109 (11)	13 (9)
Any myocardial infarction at one year	194 (8)	81 (12)	61 (6)	8 (6)
Cardiovascular death at one year	225 (9)	78 (11)	56 (6)	5 (4)
Non-cardiovascular death at one year	78 (3)	55 (8)	50 (5)	12 (9)
All-cause death at one year	303 (12)	133 (20)	106 (11)	17 (12)
**Adjudicated diagnosis of type 2 myocardial infarction**				
	Yes (n = 122)	No (n = 594)	Yes (n = 57)	No (n = 194)
Coronary angiogram at 30 days	25 (20)	57 (10)	18 (32)	21 (11)
Revascularisation at 30 days	9 (7)	9 (2)	5 (9)	2 (1)
Aspirin at 30 days	84 (69)	276 (46)	25 (44)	42 (22)
Antiplatelet therapy at 30 days	102 (84)	346 (58)	28 (49)	48 (25)
Statin at 30 days	83 (68)	353 (59)	17 (30)	44 (23)
Betablocker at 30 days	82 (67)	365 (61)	28 (49)	96 (49)
ACE inhibitor at 30 days	74 (61)	299 (50)	15 (26)	57 (29)
Cardiovascular death or MI at one year	22 (18)	76 (13)	12 (21)	14 (7)
Any myocardial infarction at one year	6 (5)	23 (4)	5 (9)	4 (2)
Cardiovascular death at one year	17 (14)	56 (9)	7 (12)	12 (6)
Non-cardiovascular death at one year	18 (15)	66 (11)	12 (21)	29 (15)
All-cause death at one year	35 (29)	122 (21)	19 (33)	41 (21)

Abbreviations: MI, myocardial infarction.

© 2024 Taggart C et al. *JAMA Network Open.*

eTable 4. Risk of subsequent myocardial infarction or cardiovascular death at one year in patients with a clinical diagnosis of myocardial infarction compared to those without a clinical diagnosis stratified by the universal definition

	Scotland	Sweden
	**Hazard ratio (95% CI)**	**Hazard ratio (95% CI)**
**Adjudicated diagnosis of type 1 myocardial infarction**		
Unadjusted	**0.69 (0.57 - 0.84)**	1.21 (0.68 - 2.16)
Adjusted for sex and age	0.84 (0.69 - 1.02)	1.21 (0.68 - 2.15)
Adjusted for conventional risk factors*	**0.77 (0.62 - 0.96)**	1.40 (0.78 - 2.51)
**Adjudicated diagnosis of type 2 myocardial infarction**		
Unadjusted	1.54 (0.96 - 2.47)	**3.58 (1.65 - 7.76)**
Adjusted for sex and age	1.52 (0.94 - 2.44)	**3.00 (1.36 - 6.60)**
Adjusted for conventional risk factors*	0.89 (0.50 - 1.58)	2.04 (0.86 - 4.87)

Conventional risk factors included age, sex, previous ischemic heart disease, previous cerebrovascular disease, diabetes mellitus, previous heart failure hospitalization, hemoglobin, myocardial ischemia, eGFR, and cardiac troponin concentrations. Abbreviations: CI, confidence interval; eGFR, estimated Glomerular Filtration rate.

© 2024 Taggart C et al. *JAMA Network Open.*

eTable 5. Clinical diagnosis of myocardial infarction using different positions in the hospital record for patients with an adjudicated diagnosis in Scotland

	Any myocardial infarction	Type 1 myocardial infarction	Type 2 myocardial infarction
**Primary position**			
Sensitivity (95% CI)	61.9% (60.4-63.4%)	72.8% (71.2-74.3%)	13.0% (10.5-15.4%)
Specificity (95% CI)	99.6% (99.6-99.7%)	99.2% (99.1-99.3%)	91.3% (90.1-91.6%)
NPV (95% CI)	94.3% (94.0-94.6%)	96.7% (96.5-96.9%)	97.6% (97.4-97.8%)
PPV (95% CI)	96.5% (95.7-97.1%)	91.7% (90.6-92.8%)	3.7% (3.0-4.4%)
Cohen's kappa	0.725	0.791	0.019
**Up to position 2**			
Sensitivity (95% CI)	66.6% (65.1-68.0%)	77.8% (76.3-79.2%)	16.1% (13.5-18.9%)
Specificity (95% CI)	99.4% (99.3-99.5%)	98.8% (98.7-99.0%)	90.5% (90.2-90.9%)
NPV (95% CI)	94.9% (94.7-95.2%)	97.3% (97.1-97.5%)	97.7% (97.5-97.9%)
PPV (95% CI)	94.6% (93.7-95.4%)	89.4% (88.2-90.5%)	4.2% (3.5-4.9%)
Cohen's kappa	0.754	0.813	0.027
**Up to position 3**			
Sensitivity (95% CI)	67.2% (65.7-68.6%)	78.4% (76.9-79.8%)	16.8% (14.2-19.6%)
Specificity (95% CI)	99.4% (99.3-99.4%)	98.8% (98.7-98.9%)	90.4% (90.1-90.8%)
NPV (95% CI)	95.0% (94.7-95.3%)	97.3% (97.1-97.5%)	97.7% (97.5-97.9%)
PPV (95% CI)	94.3% (93.4-95.1%)	89.0% (87.8-90.1%)	4.3% (3.6-5.1%)
Cohen's kappa	0.757	0.814	0.030
**Up to position 4**			
Sensitivity (95% CI)	67.3% (65.8-68.8%)	78.4% (76.9-79.8%)	17.1% (14.4-19.9%)
Specificity (95% CI)	99.3% (99.2-99.4%)	98.8% (98.7-98.9%)	90.4 (90.0-90.7%)
NPV (95% CI)	95.0% (94.8-95.3%)	97.3% (97.1-97.5%)	97.7 (97.5-97.9%)
PPV (95% CI)	94.1% (93.2-94.9%)	88.7% (87.8-90.1%)	4.3 (3.6-5.1%)
Cohen kappa	0.757	0.814	0.030
**Up to position 5**			
Sensitivity (95% CI)	67.4 (66.4 - 68.9%)	79.2 (77.2 - 80.0%)	17.1 (14.4-19.9%)
Specificity (95% CI)	99.3 (99.2 - 99.4%)	98.7 (98.6 - 98.9%)	90.4 (90.0-90.7%)
NPV (95% CI)	95.1 (94.8 - 95.3%)	97.4 (97.2 - 97.6%)	97.7 (97.5-97.9%)
PPV (95% CI)	94.0 (93.1 - 94.8%)	88.7 (87.5 - 89.8%)	4.3 (3.6-5.1%)
Cohen's kappa	0.757	0.814	0.030

© 2024 Taggart C et al. *JAMA Network Open.*

eTable 6. Additional analysis stratifying by the presence of ST-elevation on the electrocardiogram and effect on the diagnostic performance of a clinical diagnosis of myocardial infarction in the hospital record stratified by the universal definition

	**Clinical diagnosis of myocardial infarction in Scotland: non-ST-elevation myocardial infarction**		
**Adjudicated diagnosis**	Any (n = 3,266)	Type 1 (n = 2,520)	Type 2 (n = 716)
True positives	2,026	1,883	119
False positives	169	312	2,076
True negatives	24,673	25,276	25,319
False negatives	1,240	637	594
Sensitivity (95% CI)	62.0% (60.4-63.7%)	74.7%% (73.0-76.4%)	16.7% (14.1-19.6%)
Specificity (95% CI)	99.3% (99.2-99.4%)	98.8% (98.6-98.9%)	92.4% (92.1-92.7%)
Cohen's kappa	0.715	0.780	0.045
	**Clinical diagnosis of myocardial infarction in Scotland: ST-elevation myocardial infarction**		
**Adjudicated diagnosis**	Any (n = 675)	Type 1 (n = 667)	Type 2 (n = <5)
True positives	631	623	<5
False positives	169	177	797
True negatives	24,673	24,673	24,717
False negatives	44	44	<5
Sensitivity (95% CI)	93.4% (91.4-95.2%)	93.3% (91.3-95.4%)	87.5% (55.6-100%)
Specificity (95% CI)	99.3% (99.2-99.4%)	99.2% (99.2-99.4%)	96.9% (96.7-97.1%)
Cohens kappa	0.850	0.840	0.007

© 2024 Taggart C et al. *JAMA Network Open.*

eTable 7: Characteristics associated with a clinical diagnosis in populations of both ST segment elevation and non-ST-elevation myocardial infarctions in Scotland

	Odds ratio of a clinical diagnosis of myocardial infarction	
	Univariable model OR (95% CI)	Multivariable model OR (95% CI)
**Patients with non-ST-elevation myocardial infarction**		
Age (per 10 years)	0.76 (0.72-0.80)	0.94 (0.87-1.05)
Sex (women)	0.58 (0.50-0.67)	1.09 (0.92-1.29)
Ischemic heart disease (yes)	0.63 (0.55-0.74)	0.93 (0.75-1.08)
Cerebrovascular disease (yes)	0.60 (0.65-1.13)	0.85 (0.63-1.14)
Diabetes mellitus (yes)	1.28 (1.05-1.56)	1.84 (1.44-2.35)
Heart failure hospitalization (yes)	0.55 (0.45-0.66)	0.66 (0.52-0.83)
ECG ischemia (yes)	1.61 (1.38-1.87)	1.18 (0.99-1.40)
eGFR (per 10 ml/min)	1.17 (1.13-1.20)	1.16 (1.12-1.21)
Hemoglobin (per 10 mg/l)	1.14 (1.10-1.17)	1.03 (0.98-1.08)
Cardiac troponin (per log)	1.41 (1.37-1.46)	1.42 (1.37-1.46)
**Patients with ST segment elevation myocardial infarction**		
Age (per 10 years)	0.97 (0.78-1.22)	1.15 (0.87-1.51)
Sex (women)	1.61 (0.78-3.32)	2.80 (1.24-6.37)
Ischemic heart disease (yes)	0.69 (0.28-1.60)	0.89 (0.75-1.08)
Cerebrovascular disease (yes)	0.71 (0.20-2.40)	0.86 (0.22-3.35)
Diabetes mellitus (yes)	2.58 (0.61-10.9)	2.67 (0.58-12.19)
Heart failure hospitalization (yes)	0.99 (0.34-2.88)	1.17 (0.35-3.83)
ECG ischemia (yes)	1.25 (0.57-1.02)	0.89 (0.38-2.08)
eGFR (per 10 ml/min)	1.21 (1.08-1.37)	1.34 (1.15-1.55)
Hemoglobin (per 10 mg/l)	1.15 (1.06-1.25)	1.02 (0.84-1.24)
Cardiac troponin (per log)	1.41 (1.37-1.45)	1.16 (1.06-1.27)

© 2024 Taggart C et al. *JAMA Network Open.*
