# Supplementary material for: Application of the Universal Definition of Myocardial Infarction in Clinical Practice in Scotland and Sweden
Source: JAMA Netw Open. 2024 Apr 8;7(4):e245853. doi: 10.1001/jamanetworkopen.2024.5853 (PMC11002705; doi:10.1001/jamanetworkopen.2024.5853)
Supplement: Supplement 2. — Data Sharing Statement [file jamanetwopen-e245853-s002.pdf]

# Data Sharing Statement

Taggart. Application of the Universal Definition of Myocardial Infarction in Clinical Practice in Scotland and Sweden. *JAMA Netw Open*. Published April 08, 2024.
doi:10.1001/jamanetworkopen.2024.5853

**Data**
**Data available:** Yes
**Data types:** Deidentified participant data
**How to access data:** https://dataloch.org/
**When available:** With publication

**Supporting Documents**
**Document types:** None

**Additional Information**
**Who can access the data:** Anyone requesting the data with the necessary governance training and approvals
**Types of analyses:** Research or validation
**Mechanisms of data availability:** With investigator support and approval for proposal
**Any additional restrictions:** Data is stored in a NHS safe haven and can be accessed remotely
